# Right posterior petrosectomy for resection of petroclival meningioma

**DOI:** 10.3171/2022.1.FOCVID21227

**Published:** 2022-04-01

**Authors:** Kunal V. Vakharia, Ryan M. Naylor, Hirotaka Hasegawa, Ashley M. Nassiri, Colin L. W. Driscoll, Michael J. Link

**Affiliations:** 1 Department of Neurosurgery, Mayo Clinic, Rochester, Minnesota;; 2 Department of South Florida, University of South Florida, Tampa, Florida;; 3 Department of Neurosurgery, University of Tokyo, Bunkyo, Tokyo, Japan; and; 4 Department of Otorhinolaryngology, Mayo Clinic, Rochester, Minnesota

**Keywords:** petroclival meningioma, posterior petrosectomy, adjuvant radiosurgery

## Abstract

Petroclival meningiomas, which arise from the upper two-thirds of the clivus and are medial to the trigeminal nerve, carry significant surgical risk. Patients whose operations are tailored to maximize tumor resection while minimizing neurological morbidity have favorable outcomes. Subtotally resected tumors can be subsequently considered for radiosurgery in an attempt to limit recurrence. Here the authors report the case of a 40-year-old woman with postpartum trigeminal neuropathy secondary to a petroclival meningioma. The patient underwent an aggressive subtotal resection via a posterior petrosal approach with preservation of neurological function followed by adjuvant radiosurgery.

The video can be found here: https://stream.cadmore.media/r10.3171/2022.1.FOCVID21227

## Transcript

Here we present a case of a right posterior petrosectomy for resection of a petroclival meningioma

### 0:25 History of Present Illness and Physical Examination.

The patient is a 40-year-old female who began noticing tingling and numbness in her right cheek and jaw shortly after the delivery of her second child. She had been tolerating this well for 2 months prior to presentation until she developed throbbing headaches. On exam the patient had reduced pinprick sensation in V3 by 5% on formal pinprick testing and otherwise normal function. Her audiogram was normal with 100% word recognition.

### 0:48 Neuroimaging Findings.

Axial T1 contrasted imaging demonstrated a 3-cm mass that was contrast enhancing along the petroclival junction eccentric to the right. T1 sagittal and coronal imaging also demonstrates the tumor extending with its superior portion all the way up to the peduncle. Here we have axial FIESTA imaging. The image on the left demonstrates a blue arrow that highlights the internal auditory canal, and the image on the right demonstrates a green arrow demonstrating exactly where the vertebral artery on the right is and where the basilar artery is in relation to the tumor.

### 1:26 Rationale for Procedure and Surgical Approach.

Surgical intervention was chosen because of the patient’s young age and progression of symptoms. Although her symptoms may have been exacerbated by the hormonal fluctuations, patients who have progressive symptomatology and growth in tumors typically progress. Serial imaging and radiosurgery were options, but given the sensitivity of neurostructures in the area, radiosurgery in an isolated fashion was not thought to be the best option. This image shows a blue triangle that demonstrates an isolated middle fossa approach, an orange triangle that demonstrates an isolated retrosigmoid approach, and a green triangle that demonstrates a combined posterior petrosectomy approach. We do our approach in one sitting for a combined posterior petrosectomy.

### 2:03 Description of Setup.

The patient was positioned in a lateral decubitus position with the head in a Mayfield head holder.

### 2:11 Necessary Equipment.

An operative microscope and neuromonitoring specifically for cranial nerves V, VI, VII, VIII, X, and XI were used.

### 2:18 Key Surgical Steps.

The scalp and muscle were mobilized as separate flaps prior to the craniotomy to allow for better cosmesis upon closure. The craniotomy consists of a temporo-occipital craniotomy over the distal transverse sinus with large, troughed burr holes over the sinus to allow for adequate dural separation. A transmastoid approach to the presigmoid dura, which is retrolabyrinthine, opens the surgical corridor to access the higher portion of the tumor as well as to allow for easier and safer division of the tentorium. The insertion of the vein of Labbé into the dural venous sinus should be continuously searched for to avoid inadvertent injury to the critical venous structures. Finally, in addition, some surgeons use a lumbar drain for brain relaxation, and we believe in opening cisternal fluid to allow for sufficient brain relaxation for this procedure.

### 2:59 Mastoidectomy After Craniotomy.

After the craniotomy, a retrolabyrinthine mastoidectomy is performed. Here we are using a cutting burr to go through the mastoid air cells and the mastoid antrum. This is done to skeletonize the sigmoid sinus. The sigmoid sinus is decompressed all the way down to the jugular bulb.

### 3:15 Drilling Trautmann’s Triangle and Opening the Posterior Fossa Dura.

We drilled Trautmann’s triangle using a diamond burr for this situation. We drilled the rest of the mastoid down until we see a normal dural surface. CSF can be released from the cochlear aqueduct to help with cerebellar relaxation. We complete drilling Trautmann’s triangle and exposing posterior fossa dura. The sigmoid sinus is protected with Surgicel. The posterior fossa dura is opened in a curvilinear fashion. This dura is tacked up anteriorly, and the cisterns are opened to allow for sufficient cerebellar relaxation.

### 4:06 Opening Middle Fossa Dura and Cutting the Tentorium.

Similarly, we then open the middle fossa dura. The middle fossa dura is dissected medially until the edge is identified. We then clip the superior petrosal sinus. This is then sharply divided and the tentorium is divided all the way to the medial edge. During this process we make sure to visualize the trochlear nerve for the entirety of the dissection.

### 4:41 Tumor Resection.

Arachnoid dissection and tumor biopsy are performed early in the procedure. We start by wide arachnoid dissection within the posterior fossa to allow for sufficient access to all borders of the tumor. This is then followed by an early biopsy of the tumor so we can get frozen pathology intraoperatively. Here you can see us taking a biopsy just above cranial nerve number V. The arachnoid is dissected all the way back to the origin of the trigeminal nerve given the patient’s symptoms. And the surgical corridor between cranial nerve V and VII is then opened up and tumor is dissected off of the edge of the cerebellum. Early cautery of the tumor capsule allows for early internal debulking of the tumor, which allows for manipulation of the tumor capsule. This allows for safer dissection around cranial nerves. An ultrasonic aspirator is used to internally debulk the tumor for easier manipulation of the tumor capsule. Here you can see us working along the tumor capsule and bipolaring it, and then this is also resected using an ultrasonic aspirator. We then work toward mobilizing and debulking the superior edge of the tumor. Internal debulking of all compartments of the tumor, particularly the superior end, allows for easier manipulation of the tumor and tumor capsule away from surrounding cranial nerves. Here we dissect the tumor capsule off the adjacent brainstem and cerebellum. We stimulate the facial nerve and then debulk the tumor between the trigeminal nerve and vestibulocochlear complex. Here we inspect the trigeminal nerve and ensure adequate decompression. Here we can see the motor root of the trigeminal nerve. The tumor is then debulked from underneath the trochlear nerve, working within the trochlear and trigeminal corridor. The infratrochlear portion is then coagulated and sharply divided, and then this portion of the tumor is then dissected and taken out. We then focus our attention to debulking the tumor above the trochlear nerve, initially by dissecting the arachnoid off of the tumor and then coagulating the tumor capsule to allow us to create a corridor for internal debulking. We incise the tumor in a linear fashion and internally debulk the tumor for better manipulation of the tumor and tumor capsule. Using microdissectors, we were able to pull the tumor inferiorly and then coagulate the tumor capsule to allow us to be able to resect it safely from within our surgical corridor. This portion of the tumor that is supratrochlear is then debulked, and the capsule is sharply dissected from the posterior margin and resected. The oculomotor nerve and cerebral peduncle are visualized at the top end of the tumor. Here we retract the tumor capsule along the superior margin, and as we are pulling down you can clearly visualize the cerebral peduncle. This portion of the tumor is debulked using an ultrasonic aspirator as well. Then our attention focuses on the fourth cranial nerve and dissecting it off of the tumor capsule. A papaverine-soaked Gelfoam is placed along the trochlear nerve, and the tumor along the trochlear nerve corridor is then debulked using the ultrasonic aspirator. After tumor resection, the facial nerve is reinspected to confirm that no inadvertent injury occurred.

### 9:02 Closure.

Dural sealant was used to protect the surgical site. A small piece of temporalis fascia was used to cover the antrum of the middle ear. The remaining mastoid air cells were covered in bone wax and the middle fossa dura was reapproximated with 4-0 Nurolon sutures. The remainder of the defect, including the mastoidectomy defect, was filled with autologous fat graft from the abdomen.

### 9:23 A Brief Review of Clinical and Imaging Outcomes.

Six months postoperatively the patient was doing well with resolution of all of her trigeminal pain. She had no cranial neuropathies, and her audiogram demonstrated good hearing with 100% word recognition. T1 axial contrast imaging demonstrates a small residual tumor along the petroclival junction with adequate decompression of the cerebral peduncle and brainstem. In addition, sagittal and coronal imaging demonstrates that there is a small rind of tumor that is a much smaller radiosurgical target.

### 9:49 Learning Points.

Learning points from this case include preservation of cranial nerve function being paramount when resecting petroclival meningiomas. Adjunct radiosurgery can be a good option to manage these tumors long-term and preserve patient quality of life. A posterior petrosal approach allows for sufficient visualization from the jugular foramen up to the premedullary cistern. The additional exposure of the presigmoid, retrolabyrinthine dura allows for maneuverability to access the higher portions of the tumor.

### 10:15 References^1–5^

## Disclosures

The authors report no conflict of interest concerning the materials or methods used in this study or the findings specified in this publication.

## Author Contributions

Primary surgeon: Link, Driscoll. Assistant surgeon: Vakharia, Hasegawa, Nassiri. Editing and drafting the video and abstract: Link, Vakharia, Naylor, Nassiri. Critically revising the work: Link, Vakharia, Naylor, Nassiri, Driscoll. Reviewed submitted version of the work: all authors. Approved the final version of the work on behalf of all authors: Link. Supervision: Link.
